# Observational study of selective screening for prediabetes and diabetes in a real-world setting: an interprofessional collaboration method between public dental services and primary health care in Sweden

**DOI:** 10.1080/02813432.2023.2299114

**Published:** 2024-02-07

**Authors:** Katri Harcke, Anders Lindunger, Erik Kollinius, Mihretab Gebreslassie, Anna Ugarph Morawski, Charlotta Nylén, Magnus Peterson, Tülay Yucel-Lindberg, Claes-Göran Östenson, Pia Skott, Nouha Saleh Stattin

**Affiliations:** a Department of Neurobiology, Care Sciences and Society, Division of Family Medicine, and Primary Care, Karolinska Institutet, Huddinge, Sweden; b Academic Primary Health Care Centre, Stockholm, Sweden; c Department of Dental Medicine, Karolinska Institutet, Sweden; d Public Dental Services, Region Stockholm, Sweden; e Centre for Epidemiology and Community Medicine, Region Stockholm, Sweden; f Department of Global Public Health, Karolinska Institutet, Stockholm, Sweden; g Department of Public Health and Caring Sciences, Section General Medicine, Uppsala University, Sweden; h Academic Primary Health Care, Region Uppsala, Sweden; i Department of Molecular Medicine and Surgery, Endocrine and Diabetes Unit, Karolinska Institutet, Karolinska University Hospital, Solna, Sweden

**Keywords:** Diabetes mellitus type 2, oral health, periodontitis, primary health care, prediabetic state, noncommunicable disease

## Abstract

**Objective:**

Describe a method in a real-world setting to identify persons with undiagnosed prediabetes and type 2 diabetes through an interprofessional collaboration between Public Dental Services and Primary Health Care in Regions Stockholm.

**Design:**

A descriptive observational study.

**Setting:**

The study was conducted at seven sites in the region of Stockholm, Sweden. Each collaborating site consisted of a primary health clinic and dental clinic.

**Subjects:**

Study participants included adults over 18 years of age who visited the Public Dental Services and did not have a medical history of prediabetes or type 2 diabetes.

**Main outcome measures:**

Selective screening is conducted in accordance with a risk assessment protocol at the Public Dental Services. In the investigated method, DentDi (Dental and Diabetes), adults diagnosed with caries and/or periodontitis over a cut-off value are referred to the Primary Health Care clinic for screening of prediabetes and type 2 diabetes.

**Results:**

DentDi, introduced at seven sites, between the years 2017 and 2020, all of which continue to use the method today. A total of 863 participants from the Public Dental Services were referred to the Primary Health Care. Of those 396 accepted the invitation to undergo screening at the primary health care centre. Twenty-four individuals did not meet the inclusion criteria, resulting in a total of 372 persons being included in the study. Among the 372 participants, 27% (101) had elevated glucose levels, of which 12 were diagnosed with type 2 diabetes and 89 with prediabetes according to the study classification.

**Conclusions:**

DentDi is a feasible method of interprofessional collaboration where each profession contributes with the competence included in everyday clinical practice for early identification of persons with prediabetes and type 2 diabetes with a complete chain of care. The goal is to disseminate this method throughout Stockholm County and even other regions in Sweden.

## KEY POINTS

Type 2 diabetes and poor oral health have a bidirectional association. The number of persons with undetected prediabetes and type 2 diabetes is high and rising globally.

Through collaboration between Public Dental Services and Primary Health Care we have developed a feasible and novel method of selectively screening for prediabetes and type 2 diabetes in a real-world setting.

By utilizing everyday practice within each discipline, this method has been implemented at seven sites in Region Stockholm.

From the original number of 863 participants referred from the Public Dental Services to Primary Health Care 396 attended the medical screening. After excluding 24 participants, a total of 372 participants underwent screening for prediabetes and type 2 diabetes.

The results of this study showed that almost 30% who were screened for prediabetes and type 2 diabetes had elevated blood glucose levels.

## Introduction

The mouth is an important part of the body and the impact of oral health on medical health is often overlooked. Evidence-based research conducted by the World Health Organization (WHO) indicates that most oral diseases, like periodontitis and dental caries, share modifiable risk factors that can be mitigated [1]. Risk factors, such as tobacco use and an unhealthy diet, that are common to major noncommunicable diseases, can be managed to prevent adverse oral health conditions [1]. Type 2 diabetes is known to be bi-directionally associated with periodontal disease [2]. In addition, there is a causal link between the prevalence of dental caries, the most common noncommunicable disease, high sugar intake, and diabetes. The bidirectional association between type 2 diabetes and periodontitis is well known and several publications show an increased incidence of periodontitis in people with type 2 diabetes as compared to healthy controls. Similarly, people with type 2 diabetes have an increased risk of developing periodontitis [2–6]. Whereas the association between type 2 diabetes and dental caries is less explored and expected to be multifactorial [7]. This may indicate that the dental clinics are important settings for screening and early detection of prediabetes and type 2 diabetes [1]. In Sweden, the National Board of Health and Welfare has stated that dental care is important to prevent poor health and that good oral health is important for quality of life [8]. Therefore, dental care providers need to work together with other actors in the health care system.

Type 2 diabetes is a serious public health concern, and complications pose a major health threat with high costs to both the person and health care providers. Frequently asymptomatic in early stages, type 2 diabetes can remain undetected in a person until symptoms are exacerbated. While there are different recommended methods for screening, currently there is a lack of consensus on a standardized method for screening [9]. Earlier onset of complications can even begin to develop at the prediabetes stage [10]. Prediabetes, a reversible condition, is defined as having blood glucose levels higher than normal, but below the diagnostic threshold for type 2 diabetes. In Sweden, an estimated one in three cases of type 2 diabetes are undiagnosed [11], and the total prevalence is 5–6% [12–13]. Complications of type 2 diabetes are neuropathy, nephropathy, retinopathy as well as cardiovascular, renal diseases and cancer, thus increasing the risk of mortality and morbidity [14].

Periodontitis is a chronic inflammatory disease affecting the supporting tissue of the tooth [5]. The disease affects almost 40% of the population with 8% of people experiencing severe periodontitis [15–16]. Symptoms of chronic inflammation in periodontitis are due to an imbalance in the complicated relationship between oral bacteria, the hosts’ immune system, genetics, and factors that depend on behavior, environment, and lifestyle. Risk factors for periodontitis include inherited predisposition, smoking, and unknown or poorly controlled diabetes [5].

Dental caries is the world’s most common chronic, noncommunicable disease, affecting over 2.8 billion people in their lifetime [17]. It is a biofilm-mediated, diet-modulated, multifactorial disease resulting in a net mineral loss of dental hard tissue that forms a cavity. Dental caries is determined by biological, behavioral, psychosocial, and environmental factors.

The association between type 2 diabetes and impaired oral health provides an opportunity to selectively screen persons with increased risk for periodontitis and dental caries for prediabetes and type 2 diabetes during routine dental visits [18]. In Sweden, people from three years of age are regularly called to receive routine dental care, free of charge up to 23 years of age. About seventy percent of the Swedish population visits the dentist regularly, based on the risk to their dental health [19]. This creates an opportunity to identify persons at risk at an earlier stage, as seen in other studies. [20] Large-scale intervention studies have shown that type 2 diabetes can be delayed and even prevented [21–23] if identified early. Therefore, the method DentDi, with a complete chain of care between public dental service and primary health care, was developed.

### Aim

The aim of this study is to describe a method in a real-world setting to identify persons with undiagnosed prediabetes and type 2 diabetes, through an interprofessional collaboration between Public Dental Services and Primary Health Care in Regions Stockholm.

## Materials and methods

### Design and setting

A descriptive observational study of a screening method in a real-world setting.

This study used convenience-based nonprobability sampling to identify study sites in the region of Stockholm. Persons participating in the study met inclusion-exclusion criteria based on consideration of age, risk assessment, and past medical history.

### Participants and procedure

The Region of Stockholm consists of a total 60 Public Dental Services Clinics (PDSCs) and approximately 240 Primary Health Care Centres (PHCCs) distributed throughout different parts of the region. The DentDi method was initiated in 2017 at the first site where other types of collaborations had already been established between dentistry and primary health care [24]. By 2020, DentDi was expanded to seven locations based on proximity, varying socio-economic status, and the interest of their operational managers.

The included PDSCs and PHCCs were contacted, and meetings were scheduled. Information about the project and the screening process were presented and discussed during the meetings. To maintain the structure of the DentDi method, two facilitators (AL and KH) held regular meetings, in the beginning once a month for 30 min, and thereafter three to four times per year, with the collaborating partners (PDSCs and PHCCs). The two facilitators, a dentist from PDSC and a nurse with specialist training in diabetes care from PHCC, provided support as needed. Participating staff from all the clinics, both PDSC and PHCC, attended the meetings.

During the COVID-19 pandemic, meetings were conducted digitally, and the meeting time lasted from 15 to 30 min. They were carried out online for reasons of efficiency, time savings and health safety. Additional meetings were scheduled for information follow-ups, instructions, and updates about the method status, if required. For example: staff turnover or for pep talk. A seminar was held to share the preliminary study results with all the participating staff. This seminar was conducted for the first four participating units.

### The DentDi interprofessional collaboration method

The DentDi method involves the screening and early detection procedure of periodontal disease or caries as part of a standard, routine assessment in a clinical dental setting. Persons considered at risk, based on oral health examination, for prediabetes or diabetes type 2 are referred by the dental practitioner (or PDSC) to the Primary Health Care clinic for blood sampling and testing of HbA1C and fasting plasma glucose (FPG) levels. The analysis of glucose results determines the course of treatment if the person has prediabetes or type 2 diabetes. Tests at the primary health clinic are followed up with an evaluation and a response with results of clinical findings is sent to the referring dental clinic. The collected data are stored securely for further analysis of statistical significance.

### Oral health examination at the public dental services

In clinical practice in Sweden, all persons examined at the dental office undergo a risk assessment according to an established protocol. This examination in PDSCs of Region Stockholm is done by a dentist or a registered dental hygienist. The protocol contains four categories: general risk (A), caries risk (C), periodontal risk (P), and technical risk (T). The first three are used to determine the risk for disease in the oral cavity and the fourth, technical risk, is based on the general status of the teeth. The risk-assessment tool uses a four-graded scale, 0–3. In this study, only persons with C and P values of 2 or higher on the scale were included for further evaluation of prediabetes or type 2 diabetes at the PHCC.

The cutoff criteria for periodontal risk categorized as P2 or more consists of: 3 or more pockets >4 mm, horizontal bone loss with ≤ 1/3, 1–2 teeth with furcation involvement or vertical bone loss, bleeding on probing, and > 2 mm radiographic bone loss. The cutoff criteria for caries risk categorized as C2 or more consists of 1–2 new caries lesions or progression of initial caries lesions, severe damage of the enamel from erosion <1/3 of the dental enamel eroding on the buccal surface, and occlusal surface or at the lingual surface.

The inclusion for the study criteria was: 18 years of age or older, a basic understanding of oral or written Swedish (or with the assistance of a hired interpreter), a complete dental examination performed by a dentist or dental hygienist ranked with a risk assessment of C2, C3, P2 or P3. The exclusion criteria were previously known prediabetes or diabetes. If the person met the inclusion criteria, information about the study was provided and an invitation for participation in the study was made. Upon approval of participation in the study, a referral to the PHCC was issued.

### Diabetes risk assessment at the primary health care

**Figure 1.**
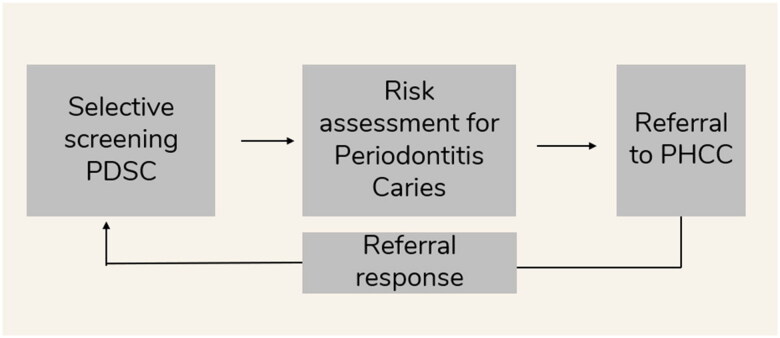
Schematic illustration of the DentDi method, a complete chain of care.

Upon receiving the referral for the person, the PHCC continued the process by contacting the person to book an appointment. At the first appointment, the person provided written consent of their participation in the DentDi method. When written consent was obtained, the Finnish diabetes risk score test (FINDRISC) was administrated [25]. Blood samples for HbA1c and fasting plasma glucose (FPG), were collected at the PHCCs laboratory. According to clinical parameters, if fasting glucose was 6.1–6.9 mmol/l and/or HbA1c 39-47 mmol/mol, an oral glucose tolerance test (OGTT) was performed at the PHCC laboratory, in accordance with Swedish guidelines. [26]. A referral response with clinical findings was sent to the dental clinic. (Figure 1)

At the start of the DentiDi method in 2017, we based our classification in accordance with the Region Stockholm guidelines. The guidelines were revised in 2019 [27] in accordance with WHO criteria [28] from HbA1c 39-47 to HbA1c 42-47 mmol/mol. We kept the reference interval for HbA1c as it was classified at the start of the study.

The normal glucose group had blood glucose as follows: FPG 4-6 mmol/L and HbA1c ≤38 mmol/mol. Whereas, the elevated glucose group had blood glucose: FPG 6,1 mmol/L or higher venously, and/or HbA1c 39 mmol/mol or higher, and/or OGTT 7,8 mmol/L, or higher or 8,9 mmol/L or higher capillary.

Prediabetes was defined as FPG 6,1-6,9 mmol/L and/or OGTT capillary 8,9–12,1 mmol/L, or venously 7,8 mmol/L- 11,0 mmol/L and/or HbA1c 39-47 mmol/mol [27, 28]. Type 2 diabetes was defined according to the guidelines for diagnosing diabetes [10, 27, 28].

The informed consents, the referral responses and the FINDRISC results were coded with a study number in a password-protected study key document to keep the data set anonymous.

### Statistical analysis

All survey datasets were then transferred to Microsoft Excel and analyzed using the statistical program R version 4.0.2. The descriptive analysis of the material was made with frequency and percentage for categorical variables and mean and standard deviation for the continuous variables. The process of the DentDi method is also qualitatively described.

### Ethical approval

The study was approved by the ethical board in Stockholm Dnr: 2013/2303-31/3.

## Results

### DentDi method

The DentDi method continues to be used at all the seven sites in the region of Stockholm, with successful adherence to procedural steps. There was a reduction in the number of required meetings due to increased efficiency between the collaborating clinics. The role of the appointed key persons at the meetings; a dentist, a registered dental hygienist, diabetes nurse, and managers served to discuss challenges and opportunities for cooperation with the participating staff. The meetings between participating clinics and staff improved the chain of care from patient screening to diagnosis and data collection of person results. The transition to digital meetings after the pandemic has reduced the need for physical meetings. This has resulted in a reduced importance of proximity between PDSCs and PHCCs identified at the beginning of the study.

While local adjustments were made to facilitate the process in daily clinical practice, no significant changes were made to the original DentDi method.

### Characteristics of the study samples and response rate

**Figure 2.**
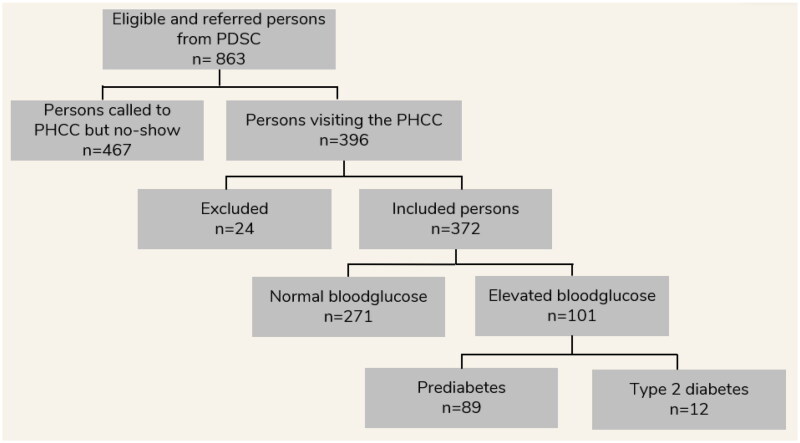
Flowchart of included participants in DentDi.

Data was collected during the period between January 2017 and February 2020. Total referrals from the included sites were obtained from 863 persons and of those, 396 accepted the invitation to get screened for type 2 diabetes at the PHCC, and to participate in the study. Twenty-four did not meet the inclusion criteria, and a total of 372 persons were included in the study (Figure 2).

Of the 24 participants that were excluded from the study; 14 did not go through the complete blood samplings, seven did not meet the inclusion criteria for periodontitis and/or caries, one participant was under the age of 18, one participant had already been diagnosed with diabetes and one did not sign the consent form.

A total number of 101 (27%) of the 372 included persons had blood glucose levels above normal. Of these, twelve persons were diagnosed with type 2 diabetes and 89 fulfilled the criteria for prediabetes in this study (Table 1). Of the participants that were referred to the PHCCs, 35 had exclusively caries, 258 had exclusively periodontitis and 69 participants (18.5%) in the study had both periodontitis and caries.

**Table 1.** Characteristics Of the study population: DentDi 2017 to 2020 (*N =* 372).

	Caries (C)	Periodontitis (P)	Both C and P	Total
	*N = 35*	*N = 268*	*N = 69*	*N = 372*
Diagnosis at PHCC:				
Normal	29 (82.9%)	186 (69.4%)	56 (81.2%)	271 (72.8%)
Prediabetes	6 (17.1%)	70 (26.1%)	13 (18.8%)	89 (23.9%)
Diabetes	0 (0.00%)	12 (4.48%)	0 (0.00%)	12 (3.23%)
Sex:				
Women	25 (71.4%)	152 (56.7%)	34 (49.3%)	211 (56.7%)
Men	10 (28.6%)	116 (43.3%)	35 (50.7%)	161 (43.3%)
Age: mean (SD)	43.2 (16.4)	52.0 (15.8)	47.4 (16.3)	50.3 (16.2)
Age:				
< 45	19 (54.3%)	90 (33.6%)	36 (52.2%)	145 (39.0%)
45-54	7 (20.0%)	61 (22.8%)	9 (13.0%)	77 (20.7%)
55-64	5 (14.3%)	45 (16.8%)	9 (13.0%)	59 (15.9%)
>64	4 (11.4%)	72 (26.9%)	15 (21.7%)	91 (24.5%)
BMI:				
< 25	14 (43.8%)	83 (31.1%)	28 (41.8%)	125 (34.2%)
25–30	10 (31.2%)	117 (43.8%)	17 (25.4%)	144 (39.3%)
> 30	8 (25.0%)	67 (25.1%)	22 (32.8%)	97 (26.5%)
Findrisc:				
Low risk (<12)	19 (76.0%)	121 (64.4%)	28 (62.2%)	168 (65.1%)
High risk (> =12)	6 (24.0%)	67 (35.6%)	17 (37.8%)	90 (34.9%)

SD: standard deviation.

More women (56.7%) than men (43.3%) participated in the study. The mean age in the study group was 50.3 years. The group with caries had the lowest mean age, 43.2 years (Table 1).

Of those who completed FINDRISC questionnaire 258 participants (34.9%) had a high risk (≥12 points).

## Discussion

The DentDi method brings together the public dental services with the primary health care to provide optimized care and treatment. The method is based on already existing everyday routines where Public Dental Services risk assess the oral health and primary care is responsible for blood glucose testing and diagnosis. Bringing together these two aspects of each medical practice is the fundamental element that demonstrates the attainability and efficacy of the DentDi method. This zipper-like approach where each discipline does what they are trained for, encourages feasibility. In this study, a total number of 372 persons were included and 27% had elevated blood glucose values. Based on the results of the study, from both the data and clinical practitioners’ perspectives, the DentDi method seems to be a feasible method for early identification of people with prediabetes and type 2 diabetes by interdisciplinary collaboration between PDSCs and PHCCs.

### Findings in relation to other studies

Previous studies of screening for diabetes type 2 in dental practice have included screening for risk factors such as age, BMI [7, 29–31] combined with oral health (preferentially periodontitis). Conducted only at the PDSCs, these screening methods have been challenging to implement in PDSCs due to limited knowledge, resources, and time. The DentDi method described in this study uses the advantage of the everyday routines which we believe is an important component for gaining feasibility in everyday practice. By risk assessment based on a validated protocol established in PDSCs, people at risk of pre-diabetes and type 2 diabetes are identified for further investigation *via* PHCC. We believe that the high diagnostic precision, 27%, seen in this study is a consequence of this feasibility. To the best of our knowledge, this is the first study on selective screening for prediabetes and type 2 diabetes describing a diagnostic method with a complete chain of care engaging PDS and PHC.

### Limitations and strengths

A limitation of this study was the non-attendance of 467 persons who accepted referral to the PHCC for further examination but did not attend their first appointment at the PHCC for testing and diagnosis (Figure 2) This can introduce several limitations and challenges. Loss to follow-up can compromise the overall effectiveness of a screening intervention. When a significant number fail to attend follow-up appointments, the true impact and benefits of the screening program may be underestimated. This, in turn, can affect the program´s ability to accurately measure its outcomes and make well-informed decisions for future implementation. Consequently, we are investigating this issue in depth in an ongoing qualitative interview study with staff and participants in DentDi. The intention is to improve the screening method to address the loss to follow-up. Possible strategies could be improvement of patient education and information about the importance of follow-up, personalized reminders, improved communication, and coordination between PDSC and PHCC, and outreach initiatives to engage patients who have missed their appointments. Digital systems can serve as valuable tools in facilitating these strategies.

During the implementation process, the study protocols were further developed to include the FINDRISC, OGTT and the referrals response step to the PDSCs. These components were not part of the method design initially, however, they were later included based on requirements. While originally a limitation, we were able to improve and enhance the DentDi method by adding these steps. Contrarily, this explains some of the missing statistical data for FINDRISC.

The observed strengths of this method in practice has been the comprehensive chain of care provided to the person, with referrals between two different health services. Enhanced communication between different health services has been shown to provide higher quality follow-up for persons [32]. The diagnosis for type 2 diabetes and prediabetes was made at the PHCC where the person received information about their diagnosis and they were also contacted for a follow-up, according to standards set by Swedish national guidelines.

### Generalizability to other settings

In Sweden, further studies are needed and several Swedish regions have expressed active interest in the DentDi method. The Public Dental Services has highlighted the need for cooperation and the Swedish National Board of Health and Welfare has begun an investigation into increased cooperation between dental care and primary care.

In large parts of Sweden’s PDCs, similar oral risk assessments are used in everyday practice, indicating that generalizability is possible.

At an overall organizational level, there are aggravating factors to be mentioned. The current organizational partition between Public Dental Services and Primary Health Care in Sweden is a challenge to consider. Additionally, digital journals and referral administration systems are unlinked, which makes it difficult to transfer interprofessional information. This lack of digital bridging complicates collaborative and multidisciplinary projects such as DentDi, makes identification of persons with undiagnosed prediabetes and type 2 diabetes less effective and successful. The goal of reducing the number of people with unrecognized or diagnose type 2 diabetes and prediabetes in the region is thus made more difficult. However, with an efficient system for electronic referral, we believe this obstacle could be overcome.

## Conclusions

DentDi is a feasible method to be used in clinical practice for early identification of persons with prediabetes and type 2 diabetes. Interprofessional collaboration as in DentDi where each profession does what it is trained to do, could be the most important aspect of the feasibility for this method. The goal is to implement this method throughout Stockholm County and possibly even other regions in Sweden. Additionally, we plan to perform a health economic analysis.

Furthermore, this ongoing method might pave the way for collaboration between PDSCs and PHCCs for other public health diseases where the oral health can reveal risk at an early stage.
